# A Data Mining Approach to Improve Inorganic Characterization of* Amanita ponderosa* Mushrooms

**DOI:** 10.1155/2018/5265291

**Published:** 2018-01-31

**Authors:** Cátia Salvador, M. Rosário Martins, Henrique Vicente, A. Teresa Caldeira

**Affiliations:** ^1^HERCULES Laboratory, Évora University, Largo Marquês de Marialva 8, 7000-809 Évora, Portugal; ^2^Departamento de Quimica, School of Sciences and Technology, Évora University, Rua Romão Ramalho 59, 7000-671 Évora, Portugal; ^3^Centro ALGORITMI, Universidade do Minho, Braga, Portugal

## Abstract

*Amanita ponderosa* are wild edible mushrooms that grow in some microclimates of Iberian Peninsula. Gastronomically this species is very relevant, due to not only the traditional consumption by the rural populations but also its commercial value in gourmet markets. Mineral characterisation of edible mushrooms is extremely important for certification and commercialization processes. In this study, we evaluate the inorganic composition of* Amanita ponderosa* fruiting bodies (Ca, K, Mg, Na, P, Ag, Al, Ba, Cd, Cr, Cu, Fe, Mn, Pb, and Zn) and their respective soil substrates from 24 different sampling sites of the southwest Iberian Peninsula (e.g., Alentejo, Andalusia, and Extremadura). Mineral composition revealed high content in macroelements, namely, potassium, phosphorus, and magnesium. Mushrooms showed presence of important trace elements and low contents of heavy metals within the limits of RDI. Bioconcentration was observed for some macro- and microelements, such as K, Cu, Zn, Mg, P, Ag, and Cd.* A. ponderosa* fruiting bodies showed different inorganic profiles according to their location and results pointed out that it is possible to generate an explanatory model of segmentation, performed with data based on the inorganic composition of mushrooms and soil mineral content, showing the possibility of relating these two types of data.

## 1. Introduction

Mushrooms are known from ancient times for their medicinal properties and gastronomic properties. Therefore the consumption of edible wild-growing mushrooms has been very popular. The demand for the commercialization of edible wild mushrooms has proved to be a widely expanding business with increasing economic importance in many rural areas of some countries. In recent years, the consumption of edible mushrooms has been increasing and gaining prominence due to their gastronomic potential, also for their both organoleptic properties (texture and pleasant aroma) [1–4], their chemical composition, mineral content, and nutraceutical value [4–8]. Mushrooms are an important source of proteins, dietary fibres, and vitamins (B, C, D, E) containing low levels of sugar and fats. They can assimilate large amounts of water and minerals such as phosphorus, iron, potassium, cadmium, magnesium, copper, and zinc, due to the large area of mycelium overgrowing the surface layer of soil [9]. This mycelia network is ideally suited to penetrate and access soil pore spaces and an extensive surface area of fungal hyphae and physiology enable for many species on effective absorption and bioconcentration of various metallic elements, metalloids, and nonmetals [10]. Bioconcentration factor (BCF), the ratio of the element content in fruiting body to the content in underlying substrate, can express the ability of fungi to accumulate elements from substrate, and this capacity of the mushroom is affected by fungal lifestyle, age of fruiting body, specific species and element, and environment such as pH, organic matter, and pollution [9]. Moreover, the symbiotic relationships that some mushrooms species, namely,* A. ponderosa,* can establish with some plants of their habitats allowing the accumulation of high concentrations of some metals. Therefore, mineral content and organic composition of edible mushrooms are dependent on the species and the characteristics of the ecosystems in which they are inserted [11, 12]. Some minerals are essential elements for the correct human body function, although others may present toxicity [5, 6, 9, 11, 13, 14]. There are many species of edible mushrooms growing wild, and some of them are slightly characterized about minerals content and the potential of heavy metals bioaccumulation such as lead, mercury, cadmium, and silver [5, 13, 15–17]. On the other hand, due to the great diversity of wild mushrooms, the similarity between some species and the lack of knowledge of others may lead to intoxication when harvesting wild species, leading in some cases to death [18].

**Figure 1:**
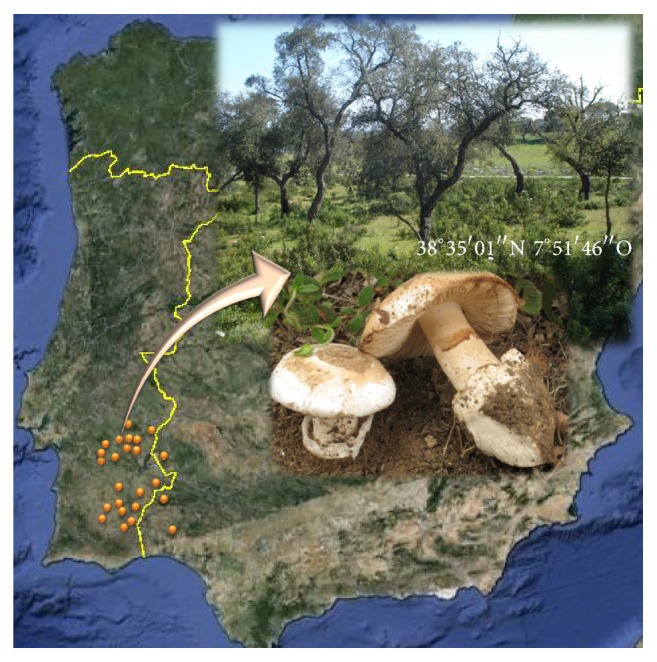
Geographic representation of sampling location areas of the* A. ponderosa* fruiting bodies of forest area of the Alentejo, Andalusia, and Extremadura regions. In detail, we observe the sampling site of Évora region (Alentejo, Portugal), showing the surrounding vegetation of* Quercus suber*,* Cistus ladanifer* and a sample of the fruiting body collected at this local.

The genus* Amanita* is one of the best known from Agaricales order and comprises edible and poisonous mushrooms distributed worldwide, occupying mainly a mycorrhizal habitat and playing a significant role in forest ecosystems [19, 20]. This genus includes important species of edible mushrooms, as is the case of* Amanita ponderosa* [18, 20–24]. The south of Portugal, due to its Mediterranean characteristics and diversity of flora, is one of the regions of Europe with a greater predominance of* A. ponderosa* wild edible mushrooms [23]. These robust basidiomata grow during spring in mounted areas with acid soils, in forests of holm oaks and cork trees like* Quercus ilex* and* Quercus suber*, and with shrubs like* Cistus ladanifer*,* Cistus laurifolius*, and* Lavandula stoechas* establishing a symbiotic relationship with them (Figure 1). Gastronomically, this species is very relevant, not only due to the traditional consumption in the rural populations, but also due to its commercial value in the gourmet markets having high exportation potential in Portugal, thus, the chemical and mineral characterization of numerous species of edible mushrooms for certification purposes and further commercialization process becoming of extreme importance.

In recent years, some artificial intelligence based tools, namely, Data Mining, Artificial Neural Networks (ANNs), and Decision Trees (DTs) have been applied for fungal environment systems [18, 25, 26]. Data mining tools were used in latest studies about* A. ponderosa* mushrooms in order to establish a bridge of inorganic contents, molecular fingerprints, and geographical sites. In one study a segmentation model based on the molecular analysis was developed, which allowed relating the clusters obtained to the geographical site of sampling. There were also developed explanatory models of the segmentation, using Decision Trees, to relate the molecular and inorganic data, following two different strategies: one based on DNA profile and another based on the mineral composition [22]. Another study based on ANNs exposed the selected model and can predict molecular profile based on inorganic composition with a good match between the observed and predicted values [18].

The aim of this study was to determine the inorganic composition of fruiting bodies of* A. ponderosa* mushrooms from different sampling sites in the southwest of the Iberian Peninsula, analysing the variations in mineral content. Additionally, the mineral composition of the corresponding soil substrates was analysed in order to correlate with the mineral composition of the fruiting bodies. In the present work, the k-means clustering method was used to study the mineral composition of* A. ponderosa* fruiting bodies. To explain the segmentation model, that is, in order to obtain rules to assign a case to a cluster DTs were used.

## 2. Materials and Methods

### 2.1. Sampling Collection

Fruiting bodies of the* Amanita ponderosa* mushrooms were collected in spring, between February and April, from 24 different sampling sites, in the southwest of the Iberian Peninsula, namely, 11 samples collected from Alto Alentejo region and 10 from Baixo Alentejo, Portugal, 2 samples collected from Andalusia and 1 from Extremadura, Spain (Table 1). These mushrooms were collected in acid soils, in forests of* Quercus suber*,* Q. ilex* ssp.* ballota*,* Cistus ladanifer, *and* Cistus laurifolius *(Figure 1).

Three individuals in the same growth stage were selected per each sampling site to avoid the effect of size. Fruiting bodies were identified by a specialist, based on morphological features according to taxonomic description of* A. ponderosa* [27]. During the collection process fruiting bodies were placed in wicker baskets and samples were transported in refrigerated containers and deposited in the Biotechnology Laboratory of Chemistry Department of University of Évora (Portugal). In parallel, soil samples were randomly collected from the surrounding soil of fruiting bodies of each site.

Fruiting bodies samples were weighed and carefully washed with double distilled water and representative samples of each sampling site were catalogued, stored in sterile bags, and preserved at −20°C for its inorganic study. The sheath was eliminated during the pretreatment of fruiting bodies samples, since it is not usually consumed.

Soil substrates were sampled (0–15 cm), after removing some visible organisms, small stones, sticks, and leaves. Samples were air-dried in room temperature for 1-2 weeks and, subsequently, sieved through a pore size of 2 mm and stored at −4°C.

### 2.2. Inorganic Characterization

Mineral composition was determined in* A. ponderosa* fruiting bodies and respective soil collected in 24 different sampling sites described, using different analytical techniques: Flame Atomic Absorption Spectrometry (FAAS), Flame Emission Photometry (FEP), and UV-Vis molecular absorption spectrometry.

#### 2.2.1. Treatment of Samples

Mineral composition analysis of fruiting bodies was performed by the dry mineralization method [28]. Three samples of mushrooms from each sampling site (25 g, fresh weight) were dried in a furnace at 100°C to constant weight (48 h) from which the moisture content was calculated. Samples were homogenized, transferred to porcelain crucibles, and incinerated in a muffle furnace (Termolab) at 460°C for 14 h. In order to determine the organic matter and mineral content in mushrooms samples, ashes were weighted at constant weight. Afterwards, ashes were bleached after cooling by adding 2 M nitric acid, drying them on thermostatic hotplates, and maintaining them at 460°C for 1 h. Ashes recovery was performed with 15 mL of 0.1 M nitric acid and stored at 4°C until analyses.

Three soil samples (5 g dw) collected from each sampling site were cold treated with an extracting solution of 25% nitric acid (HNO_3_) (40 ml) and shaking with orbital agitation for 24 h at room temperature. The extracts obtained after filtering through* Whatman* No. 42 filter paper were stored into polyethylene bottles and stored at 4°C until analyses [11].

#### 2.2.2. Analytical Determinations

Aluminium (Al), barium (Ba), calcium (Ca), cadmium (Cd), lead (Pb), copper (Cu), chromium (Cr), iron (Fe), magnesium (Mg), manganese (Mn), silver (Ag), and zinc (Zn) contents were quantified by flame atomic absorption spectrometry (Perkin-Elmer 3100 model) with atomization in an air-acetylene flame and single element hollow cathode lamps and background correction with deuterium lamp for manganese. For the determinations of calcium and magnesium, strontium chloride (SrCl_2_) was added to make up a final concentration of 0.1125% of the sample, in order to prevent anionic interferences which might modify the result [29].

Sodium (Na) and potassium (K) were quantified by flame emission photometry with a Jenway model PFP7 flame emission photometer [30, 31]. Phosphorus (P) was quantified by vanadomolybdophosphoric acid colorimetric method, using a spectrophotometer (Hitachi, U-3010 model) [32].

All sample determinations were performed in triplicate for each one of the three independent ash extracts of mushroom samples from 24 sampling sites (*n* = 9) and for three extracts of each soil substrate (*n* = 9). Concentration values were calculated through the standard curves for each element expressed in mg/kg dw. The bioconcentration factor (BCF) value, which is the quotient of the concentration in fruiting bodies divided by the concentration in the soil substrates, was determined for all minerals in each sampling site.

### 2.3. Data Mining

#### 2.3.1. Cluster Analysis

In the present study a cluster analysis was carried out. The technique applied in order to build up clusters was the k-means clustering method [33] and the software used was WEKA [34]. In WEKA Simple k-means algorithm the normalization of the numerical attributes is carried out automatically when the Euclidean distance is computed. A more detailed description of the mentioned algorithm can be found in Witten [35].

#### 2.3.2. Decision Trees

In the k-means clustering method clusters were formed without information about the groups and their characteristics. Therefore, it is necessary to understand how the clusters were formed. To attain such a purpose Decision Trees (DTs) were used. In this study the algorithm used to generate DTs was the WEKA J48 [34], corresponding to the 8th revision of the C4.5 algorithm. The detailed description of the J48 algorithm can be found in Witten et al. [35].

### 2.4. Statistical Analysis

Data were evaluated statistically using the SPSS®* 21.0* software* Windows Copyright*©,* (Microsoft Corporation)*, by descriptive parameters and by* one-way* ANOVA in order to determine statistically significant differences at the 95% confidence level (*p* < 0.05). The homogeneity of the population variances was confirmed by the* Levene* test and the multiple comparisons of media were evaluated by* Tukey's* test.

## 3. Results

### 3.1. Fruiting Bodies and Soil Substrates' Mineral Composition

Mineral analysis of* A. ponderosa* mushrooms samples collected from 24 sampling sites of Alentejo (Portugal), Andalusia, and Extremadura (Spain) regions was evaluated.

Table 2 shows the contents in moisture, organic content, and minerals present in the samples of* A. ponderosa* collected in the different places. Moisture content ranged from 89.5 ± 0.0 to 93.8 ± 0.5% (Table 2). The analyses of variance (one-way ANOVA) showed that* A. ponderosa* fruiting bodies samples collected in Almendres (1) and Serpa (20) presented significantly higher values compared to the other samples, while the sample from Mina S. Domingos (11) showed moisture content significantly lower than the remaining samples. Dry weight values, for several samples, ranged between 6.2 ± 0.5 and 10.5 ± 0.1%, consisting in organic content values between 5.8 ± 0.3 and 9.8 ± 0.1% and minerals between 0.5 ± 0.0 and 1.4 ± 0.1%. Fruiting bodies samples collected in Almendres (1) and Serpa (20) presented values of organic content significantly lower and the sample collected in the Mina S. Domingos (11) significantly higher than all analysed samples (*p* < 0.05). The mineral content of the fruiting bodies collected in Cabezas Rubias (6) was 1.4 ± 0.1%, a value significantly higher than the remaining samples. However, the samples from Serpa (20) and Villanueva del Fresno (24) showed the lowest values, significantly different from the another samples (*p* < 0.05).

**Figure 2:**
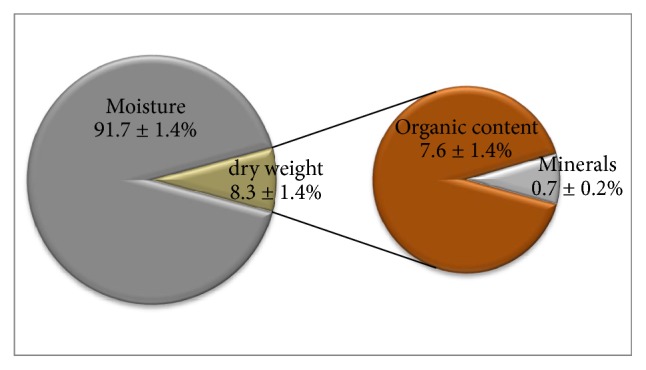
Moisture, dry weight, and organic and minerals content of* A. ponderosa* fruiting bodies from 24 different sampling sites.

Figure 2 shows the average contents of water, dry weight, organic content, and minerals present in the 24 samples of* A. ponderosa* analysed. The fruiting bodies presented water content, corresponding to 90.3 to 93.1% of their fresh weight. The organic matter content ranged between 6.2 and 9% and mineral content between 0.5 and 0.9%. Thus, 100 g of edible* A. ponderosa* mushrooms corresponds to a maximum of 9 g of macronutrients, such as lipids, carbohydrates, and proteins, and less than 1 g of mineral content. These values were similar to those observed in a study with mushrooms harvested in some regions of Andalusia (water content 87.8%, organic content 11.8%, carbohydrates 6.6%, proteins 3.2%, lipids 0.5%, fibre 1.5%, and mineral content 0.4%) [24] and these fruiting bodies have a caloric content of 42 kcal/100 g of mushroom, similar to other edible mushroom species, characterized as low calorie foods [8, 28, 36].

Tables 3 [Table tab4] [Table tab5]–6 show the mineral content of 24 sampling sites of* A. ponderosa *fruiting bodies as well as their corresponding soil samples. The studied* A. ponderosa* fruiting bodies showed higher mineral content in macroelements calcium (Ca), magnesium (Mg), sodium (Na), potassium (K), phosphorus (P), and microelements aluminium (Al), copper (Cu), and iron (Fe) (Tables 3 and 4). Potassium was present in higher concentrations and was higher in Cabezas Rubias (6) fruiting bodies, 69565 ± 362 mg/kg dw. The samples showed lower values of the trace elements silver (Ag), barium (Ba), cadmium (Cd), chromium (Cr), manganese (Mn), lead (Pb), and zinc (Zn) (Tables 5 and 6).

Minerals contents of* A. ponderosa* fruiting bodies and their corresponding soil samples presented significant differences for all the studied elements (*p* < 0.05). Cabezas Rubias (6) presented significantly higher aluminium and calcium contents (*p* < 0.05). The cadmium content was significantly higher (*p* < 0.05) in the fruiting bodies collected in N. S^ra^ Machede (16) and Almendres (1). Samples from Évora (7), Cabezas Rubias (6), and Villanueva del Fresno (24) showed similar chromium contents (*p* > 0.05), which were significantly higher than the others* A. ponderosa* fruiting bodies (*p* < 0.05). Results obtained for soil mineral content showed some significant differences between sampling sites (*p* < 0.05). The sample collected in N. S^ra^ Machede (16) presented significant different contents of silver, aluminium, and magnesium (*p* < 0.05). The Montejuntos sample (14) presented significantly differences of aluminium, iron, phosphorus, and magnesium contents (*p* < 0.05) and Villanueva del Fresno (24) presented significant differences for barium and manganese (*p* < 0.05). Significant differences in copper content were observed for Her^de^ da Mitra sample (9). Mértola sample (10) presented significant differences in zinc and Baleizão sample (3) in potassium contents (*p* < 0.05). Serpa samples (20), presented significant differences in calcium, chromium, iron, magnesium, sodium, and zinc (*p* < 0.05). Almendres (1), Évora (7), M^te^ Novo (13) and Valverde (22) samples did not present significant differences for the iron content (*p* > 0.05). Samples of Cabezas Rubias (6), Serpa (20), and Rosal de la Frontera (17) did not present significant differences in lead content (*p* > 0.05). Concerning phosphorus content, two groups with no significant differences (*p* > 0.05) were observed, one including Baleizão (3) and Rosal de la Frontera (17) and the other group including S^to^ Aleixo Restauração (18), S. Miguel de Machede (19), and Vila Nueva del Fresno (24) samples. Finally, samples from Baleizão (3) and Valverde (22) did not present significant differences in sodium and potassium content (*p* > 0.05).

**Figure 3:**
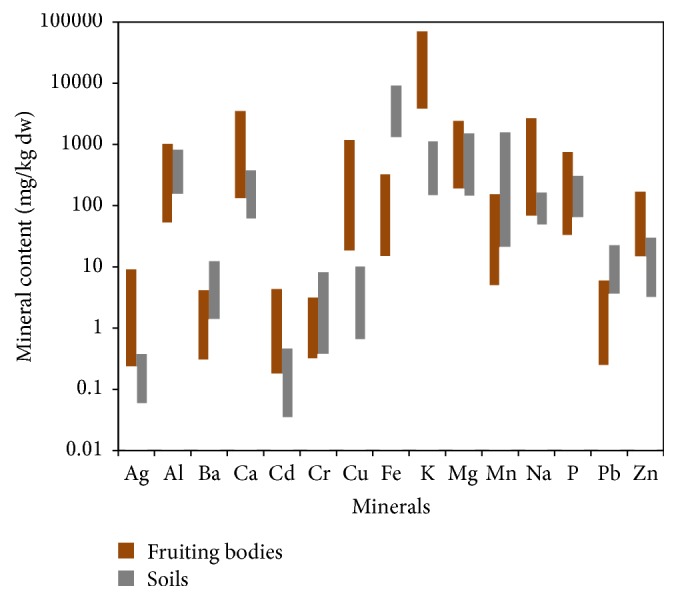
Concentration of mineral content of* A. ponderosa* fruiting bodies and soil substrates. The values are represented in logarithmic scale.

In fact, mineral content of* A. ponderosa* fruiting bodies and their soil samples (Figure 3) varied very similarly for almost all the analysed elements, indicating the influence of the substrate characteristics on the composition of mushrooms samples collected at the different sites.

Mushrooms have a specialized mechanism to accumulate nutrients and minerals in their fruiting bodies. The age of the fruiting body or its size may contribute to mineral composition. Thus,* A. ponderosa* mushrooms analysed in this study were obtained at the same development stage, in order to eliminate possible size interferences in the comparison of their mineral content. Macroelements content, such as Ca, Mg, Na, K, and P, in fruiting bodies were similar to those reported in literature for* A. ponderosa* [28] and for other edible mushrooms species [5, 36–38]. The calcium and phosphorus contents for the different samples studied were 523 ± 143 and 294 ± 171 mg/kg dw. Potassium and sodium, minerals responsible for the hydroelectrolytic balance maintenance and important enzyme cofactors, have high RDIs (Recommended Daily Intakes), which are 4700 mg/day and 2000 mg/day, respectively, for an adult [39]. Potassium and sodium levels of* A. ponderosa* mushrooms studied were 29648 ± 14908 and 1092 ± 620 mg/kg dw, respectively. Potassium content ranged from 18021 ± 1806 to 69565 ± 362 mg/Kg dw, presenting values similar to those described by Moreno-Rojas et al. [28], that report potassium levels ranging between 22410 ± 211 and 60890 ± 23950 mg/Kg dw. However, the low K levels observed for fruiting bodies from three sampling sites can be correlated with the large differences observed for potassium content in the surrounding substrates. Magnesium also plays an important role in large number of biological functions, particularly linked to energy metabolism, it is required for the proper function of certain enzymes as cofactor, and structural functions, the recommended magnesium intakes for adult (19–65 years), are 220–260 mg/day [40]. The mean magnesium content in the fruiting bodies was 738 ± 261 mg/kg dw, similar to that described in the literature [28].

*A. ponderosa* fruiting bodies presented values of trace minerals smaller than those found in other species of edible mushrooms [6, 11, 14, 38, 41]. Concentrations of trace elements in fruiting bodies are generally species-dependent [36]; however only one study of* A. ponderosa* mushrooms is reported [28]. The existence of higher metal concentrations in younger fruiting bodies can be explained by the transport of metals from the mycelium to the fruiting body during the beginning of fructification [36]. Trace elements like Fe, Cu, Zn, Cr, and Mn are essential metals since they play an important role in biological systems; however, these trace metals can also produce toxic effects when intake is excessively amount [38, 42, 43]. Copper is the third most abundant trace element found in the human body, and being an important element of several enzymes, it is also one of the agents involved in iron metabolism [42]. Iron is a component of haemoglobin and myoglobin, proteins responsible for transporting oxygen to tissues. It also participates in protein metabolism, energy production in cells and in various enzymatic reactions [44]. The copper content was 185 ± 125 mg/kg dw, and the highest value was found in* A. ponderosa* samples collected from Rosal de la Frontera (17) (584 ± 51 mg/kg dw). The iron content in* A. ponderosa* mushrooms was 92 ± 85 mg/kg dw. Copper levels were similar and iron levels are slightly lower than those described for* Amanita *spp. [5, 28]. The adult RDI is 2 mg/day for Cu and 18 mg/day for Fe [12, 39], so the concentrations of copper and iron present in these edible mushrooms are not considered to be a health risk. Manganese and zinc are important trace elements for the human organism, participating in macronutrients and nucleic acids metabolism and promoting several enzyme activity processes [14, 45, 46]. These elements can be accumulated by mushrooms and the recommended daily intakes were 2 mg/day and 15 mg/day, for manganese and zinc, respectively [12, 39]. In this study, mushrooms presented a mean value of 56 ± 27 mg/kg dw for manganese and 68 ± 25 mg/kg dw for zinc content, similar to values described in literature [5, 6, 11, 36, 47]. Chromium biological functions are not known precisely; it seems to participate in the metabolism of lipids and carbohydrates, as well as in the insulin action. The RDI for this metal is 120 *μ*g/day. The mean chromium content obtained was 1,19 ± 0,74 mg/kg dw, lower than those reported in the literature for other species of* Amanita* [5] and similar to some species of edible mushrooms described [11, 36]. Aluminium is one of the few abundant elements in nature but no significant biological function is known, although there are some evidences of toxicity when ingested in large quantities. Most acidic soils are saturated in aluminium instead of hydrogen ions, and this acidity is the result of aluminium compounds hydrolysis [48].* A. ponderosa* fruiting bodies from the different sampling sites showed a high range of aluminium content with a medium value of 362 ± 204 mg/Kg dw. High aluminium levels were reported for some* Amanita* species, for example,* A. rubescens* that showed values around 262 mg/kg dw [15] and* A. strobiliformis* and* A. verna* presented aluminium levels of 72 and 343 mg/kg dw, respectively [5]. Other studies report different levels of aluminium in* Amanita rubescens*: 293, 75, and 512 mg/kg dw for the whole fruiting body, cap, and stipe, respectively [37]. The large range in aluminium content was also reported in a study of* A. fulva* that showed aluminium levels ranging from 40 to 500 mg/Kg dw in the stipe and 40 to 200 mg/Kg dw in cap [10]. Regarding cadmium and lead, these elements have the highest toxicological significance. Cadmium has probably been the most damaging metal found in mushrooms; some studies point out the existence of accumulating species, which, in polluted areas, accumulate this metal. The mean values of cadmium and lead were 1.00 ± 0.73 mg/kg dw and 2.41 ± 1.34 mg/kg dw, respectively, showing that the* A. ponderosa* had no toxicity due to the presence of these two elements [5, 11]. The contents of heavy metals barium and silver in the studied mushrooms were 1.10 ± 0.59 mg/kg dw and 2.01 ± 1.56 mg/kg dw, respectively. These values are similar to those described in the literature for other species of edible mushrooms [11] and lower than those described for species of the genus* Amanita *[5, 13].

Bioconcentration factor (BCF) allows estimating the mushroom potential for the bioextraction of elements from the substratum (soil). Values of BCF of* A. ponderosa* fruiting bodies are summarized in Tables 7 and 8. The macroelements, K and Na, exhibited the highest values of BCF but Ca, Mg, and P also presented BCF > 1. Trace elements, Ag, Cd, Cu, and Zn, presented BCF > 1 with higher values for Cu. The remaining elements (Fe, Mn, Ba, Cr, and Pb) are bioexcluded showing lower values (BCF < 1). For copper, BCF values ranged from 9 to 248, showing bioaccumulation of this metal. The same heterogeneous behaviour is observed for Ag, Cd, and Zn with values ranging from 1–72, 1–38, to 2–25, respectively. Ag bioaccumulation was found in other* Amanita* species with much higher values of BCF [13]. The high levels of aluminium observed for some* A. ponderosa* fruiting bodies can be related to soil content and to their different ability to accumulate this mineral, with BCF > 1 for some stands. Some works with different species such as* Leccinum scabrum*,* Amanita rubescens*, and* Xerocomus chrysenteron* reported different aluminium accumulation [15]. Some species of Basidiomycetes can be useful for assessing the environmental pollution levels [38].

Metal concentrations were usually assumed to be species-dependent, but soil composition is also an important factor in mineral content [12, 36, 38, 49]. In order to clarify this association between inorganic composition of* A. ponderosa* mushrooms and soil, a data mining approach was developed.

### 3.2. Segmentation Models Based on Mushrooms and Soil Mineral Content: Interpretation and Assessment

The k-Means Clustering Method is a segmentation algorithm that uses unsupervised learning. The input variables used in the segmentation approach are macroelements content (Na, K, Ca, P, and Mg), trace elements content (Al, Fe, Cu, Zn, Cr, and Mn), and heavy metals (Ag, Ba, Cd, and Pb). The algorithm input parameter is the number of clusters, 3 in this study.  Table 9 shows the clusters centre of gravity, in order to characterize the clusters formed.

**Figure 4:**
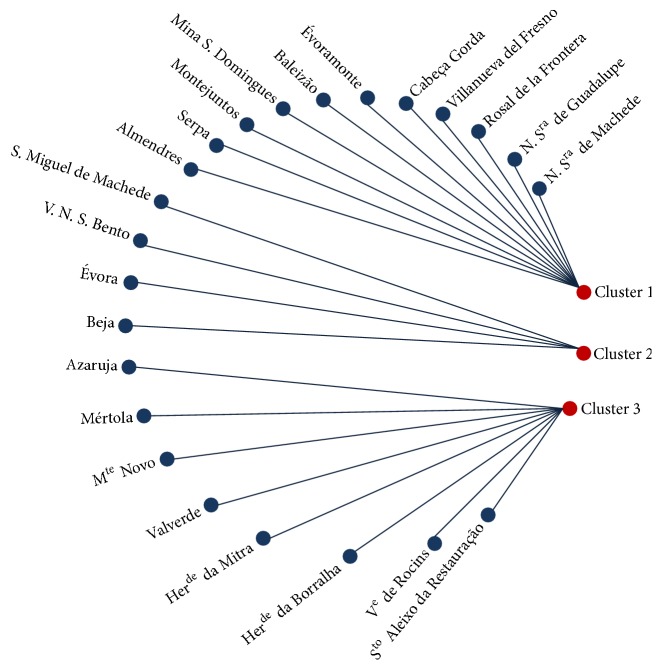
Relationships between clusters of mushrooms and different sampling sites.

The analysis of Table 9 shows that cluster 1 is characterized by high values of Ag, Ba, Cd, K, Mg, and P. Cluster 2 is characterized by high content of Cr, Fe, Mn, Na, Pb, and Zn and Cluster 3 showed lower mineral content. In order to evaluate the relationships between clusters and fruiting bodies sampling sites the graph presented Figure 4 was conceived.

The analysis of Figure 4 shows that cluster 1 is formed by the samples collected at Almendres (1), Baleizão (3), Cabeça Gorda (5), Évoramonte (8), Mina S. Domingos (11), Montejuntos (14), N. S^ra^ Guadalupe (15), N. S^ra^ Machede (16), Rosal de la Frontera (17), Serpa (20), and Villanueva del Fresno (24). Cluster 2 includes the samples collected at Beja (2), Évora (7), S. Miguel de Machede (19), and V. N. S. Bento (23). Finally, cluster 3 is composed by the samples collected at Azaruja (2), Her^de^ da Mitra (9), Mértola (10), M^te^ da Borralha (12), M^te^ Novo (13), S^to^ Aleixo da Restauração (18), V^e^ de Rocins (21), and Valverde (22).

In order to generate an explanatory model of segmentation (i.e., seek to establish rules for assigning a new case to a cluster), Decisions Trees (DT) were used. Two different strategies were followed: one of them based on the mineral mushrooms content (strategy 1) and the other one based on the soil mineral composition (strategy 2).

To ensure statistical significance of the attained results, 25 (twenty-five) runs were applied in all tests, the accuracy estimates being achieved using the Holdout method [50]. In each simulation, the available data are randomly divided into two mutually exclusive partitions: the training set, with about 2/3 of the available data and used during the modelling phase, and the test set, with the remaining examples, being used after training, in order to compute the accuracy values.

**Figure 5:**
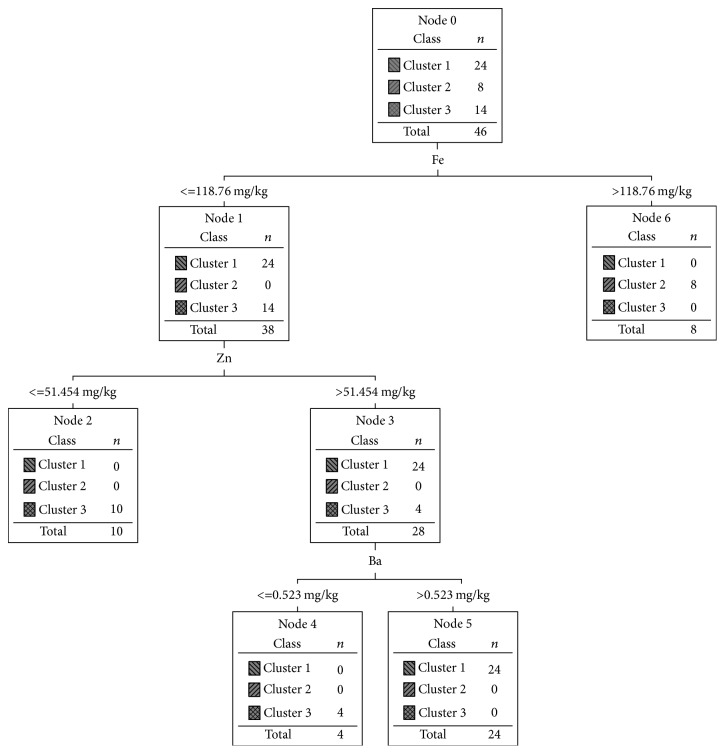
The Decision Tree explanatory of segmentation model based in mineral content of mushrooms samples (strategy 1).

The DT obtained using the strategy 1 is shown in Figure 5. The minerals that contribute to this explanatory model are Fe, Zn, and Ba and the rules to assign a case to a cluster are as follows:

(i) **If** Fe ≤ 118.76 mg/Kg
   ** and** Zn > 51.454 mg/Kg
    ** and** Ba > 0.523 mg/Kg
     ** Then** → Cluster 1
(ii) **If** Fe > 118.76 mg/Kg
 ** Then** → Cluster 2
 (iii) **If** Fe ≤ 118.76 mg/Kg
 ** and** Zn ≤ 51.454 mg/Kg
  ** Then** → Cluster 3
(iv) **If** Fe ≤ 118.76 mg/Kg
 ** and** Zn > 51.454 mg/Kg
  ** and** Ba ≤ 0.523 mg/Kg
   ** Then** → Cluster 3

A common tool for classification analysis is the coincidence matrix [51], a matrix of size *L*  ×  *L*, where *L* denotes the number of possible classes. This matrix is created by matching the predicted and actual values. *L* was set to 3 (three) in the present case.  Table 10 presents the coincidence matrix. The results reveal that the model accuracy is 100% both in the training set and in test set.

**Figure 6:**
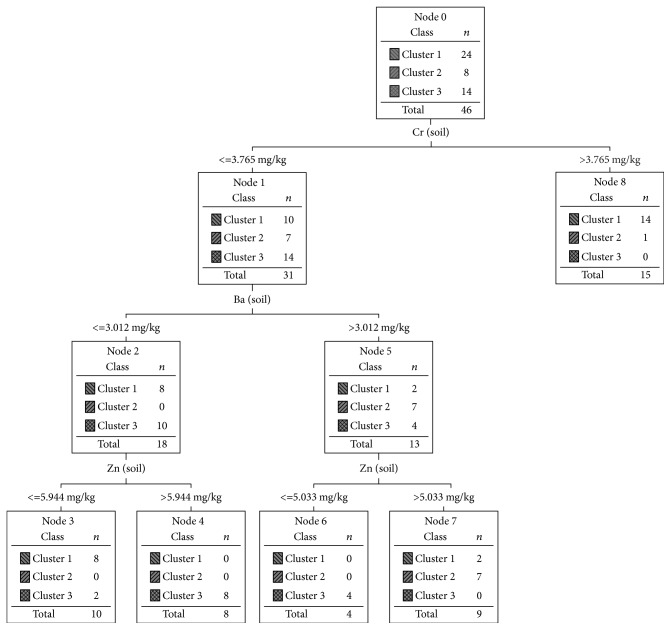
The Decision Tree explanatory of segmentation model based in mineral content of substrates.

In order to relate the mineral mushrooms content to the soil mineral composition an explanatory model of the clusters formed was made, using the soil content of the same minerals (i.e., Fe, Zn, and Ba). Since the model accuracy is only 74% a new explanation model was built up. In this attempt all inorganic soil mineral content was available. The referred model is shown in Figure 6 and the respective coincidence matrix is presented in Table 11.

The model accuracy was 89.1% and 82.6%, respectively, for training and test sets. The soil minerals that contribute to this explanatory model are Cr, Ba, and Zn. The rules to assign the cases to each cluster are as follows:

- ** If** Cr (soil) > 3.765 mg/Kg
   ** Then** → Cluster 1
- ** If** Cr (soil) ≤ 3.765 mg/Kg
   ** and** Ba (soil) ≤ 3.012 mg/Kg
    ** and** Zn (soil) ≤ 5.944 mg/Kg
     ** Then** → Cluster 1
- ** If** Cr (soil) ≤ 3.765 mg/Kg
   ** and** Ba (soil) > 3.012 mg/Kg
    ** and** Zn (soil) > 5.033 mg/Kg
     ** Then** → Cluster 2
- ** If** Cr (soil) ≤ 3.765 mg/Kg
   ** and** Ba (soil) ≤ 3.012 mg/Kg
    ** and** Zn (soil) > 5.944 mg/Kg
     ** Then** → Cluster 3
- **If** Cr (soil) ≤ 3.765 mg/Kg
   ** and** Ba (soil) > 3.012 mg/Kg
    ** and** Zn (soil) ≤ 5.033 mg/Kg
     ** Then** → Cluster 3

This study observed that the mineral content was influenced by the location area in which the mushroom samples were collected, possibly due to the soil composition and by environmental factors, such as medium temperature, vegetation, and rainfall. Indeed the inorganic composition of* A. ponderosa* allows group mushrooms according to the location area, based mainly in their Fe, Zn, and Ba content. On the other hand, it is possible to predict the same mushroom clustering taking into account the mineral soil content, based in Cr, Ba, and Zn soil composition although with a lower model accuracy.

Results of mineral composition do not reveal a direct correlation between inorganic composition of* A. ponderosa* mushrooms and their corresponding soil substrate; nevertheless, mushrooms are agents that play an important role in the continuous changes that occur in their habitats, and indeed they present a very effective mechanism for accumulating metals from the environment.

Moreno-Rojas et al. (2004), in the study of mineral content evaluation of* A. ponderosa* samples from Andalusia, also verified that variations occurred in the mineral composition according to the sample collection site, particularly in relation to Fe, K, and Na contents. Other authors also report that the main cause of variation of mineral composition between samples of different species of* Amanita* is the character and composition of the substrates (e.g., sand and wood) and may be influenced by the presence or absence of ability of the different species for a specific accumulation of some metals, namely, copper and zinc [5]. In a study carried out with a species of Boletaceae* (Suillus grevillei)*, it is also mentioned that the variations in mineral composition of the different samples are related to the composition of the substrates and the geochemistry of the soils of each site [11].

## 4. Conclusions

*A. ponderosa* mushrooms collected from different sites showed fruiting bodies with water content of 90–93%, dry mass ranging from 6.9 to 9.7%, contents of organic matter between 6.2 and 9.0%, and minerals between 0.5 and 0.9%. Mineral composition revealed high content in macroelements, such as potassium, phosphorus, and magnesium. Copper, chromium, iron, manganese, and zinc, essential microelements in biological systems, can also be found in fruiting bodies of* A. ponderosa*, within the limits of RDI. Bioconcentration was observed for some macro- and microelements, such as K, Na, Cu, Zn, Mg, P, Ag, Ca, and Cd. The presence of heavy metals, such as barium, cadmium, lead and silver, was quite low, within the limits of RDI, and did not constitute a risk to human health.

Our results pointed out that it is possible to generate an explanatory model of segmentation performed with data based on the inorganic composition of mushrooms and soil mineral content, showing that it may be possible to relate these two types of data. The inorganic analysis provides evidence that mushrooms mineral composition variation is according to the collecting location, indicating the influence of the substrate characteristics in the fruiting bodies. The relationship between mineral elements in mushrooms and soils from the different sampling sites can be an important contribution to the certification process and seem to be related to the substrate effects from interindividual or interstrain differences.

## Figures and Tables

**Table 1:** Sampling sites description of *A. ponderosa* mushrooms and soil substrates.

Sampling sites	GPS coordinates	Region/country
(1) Almendres	38°33′57′′N 8°02′40′′O	Alentejo, Portugal
(2) Azaruja	38°42′10′′N 7°45′58′′O	
(3) Baleizão	38°01′34′′N 7°42′38′′O	
(4) Beja	38°02′44′′N 7°50′56′′O	
(5) Cabeça Gorda	37°55′19′′N 7°48′47′′O	

(6) Cabezas Rubias	37°43′50′′N 7°05′11′′O	Andaluzia, Spain

(7) Évora	38°35′01′′N 7°51′46′′O	Alentejo, Portugal
(8) Evoramonte	38°46′22′′N 7°42′45′′O	
(9) Her^de^ da Mitra	38°31′35′′N 8°00′51′′O	
(10) Mértola	37°37′44′′N 7°39′30′′O	
(11) Mina S. Domingos	37°41′02′′N 7°28′49′′O	
(12) M^te^ da Borralha	37°58′31′′N 7°37′22′′O	
(13) M^te^ Novo	38°30′39′′N 7°43′06′′O	
(14) Montejuntos	38°32′24′′N 7°19′49′′O	
(15) N. S^ra^ Guadalupe	38°33′47′′N 8°01′23′′O	
(16) N. S^ra^ Machede	38°35′21′′N 7°48′19′′O	

(17) Rosal de la Frontera	37°57′21′′N 7°13′12′′O	Andaluzia, Spain

(18) S^to^ Aleixo da Restauração	38°04′01′′N 7°09′46′′O	Alentejo, Portugal
(19) S. Miguel de Machede	38°37′34′′N 7°42′33′′O	
(20) Serpa	37°55′59′′N 7°35′05′′O	
(21) V^e^ Rocins	37°52′15′′N 7°44′41′′O	
(22) Valverde	38°32′24′′N 8°01′18′′O	
(23) V. N. S. Bento	37°56′27′′N 7°23′51′′O	

(24) Villanueva del Fresno	38°22′49′′N 7°11′35′′O	Extremadura, Spain

**Table 2:** Moisture, organic content, and minerals contents of *A. ponderosa* fruiting bodies from different sampling sites.

Fruiting bodies	Composition in fresh weight		
Sampling sites	Moisture (%)	Organic content (%)	Minerals (%)
(1) Almendres	93.6 ± 0.3^a^	5.8 ± 0.3^a^	0.6 ± 0.1^a,b,c^
(2) Azaruja	92.4 ± 0.5^a,b,c,d^	6.7 ± 0.5^a,b,c^	0.9 ± 0.0^b,c^
(3) Baleizão	90.7 ± 0.3^b,c,d,e^	8.6 ± 0.4^b,c,d^	0.7 ± 0.1^a,b,c^
(4) Beja	91.0 ± 0.0^a,b,c,d,e^	8.4 ± 0.1^a,b,c,d^	0.6 ± 0.0^a,b,c^
(5) Cabeça Gorda	91.6 ± 0.7^a,b,c,d,e^	7,8 ± 0.7^a,b,c,d^	0.5 ± 0.0^a,b^
(6) Cabezas Rubias	91.8 ± 0.4^a,b,c,d,e^	6.8 ± 0.1^a,b,c^	1.4 ± 0.1^d^
(7) Évora	90.2 ± 1.6^c,d,e^	9.2 ± 1.4^c,d^	0.7 ± 0.2^a,b,c^
(8) Evoramonte	90.4 ± 0.4^b,c,d,e^	8.8 ± 0.7^b,c,d^	0.8 ± 0.3^a,b,c^
(9) Her^de^ da Mitra	90.5 ± 0.3^b,c,d,e^	8.9 ± 0.3^c,d^	0.6 ± 0.1^a,b,c^
(10) Mértola	93.2 ± 0.3^a,b^	6.0 ± 0.3^a,b^	0.8 ± 0,1^a,b,c^
(11) Mina S. Domingos	89.5 ± 0.0^e^	9.8 ± 0.1^d^	0.8 ± 0.1^a,b,c^
(12) M^te^ da Borralha	91.5 ± 1.5^a,b,c,d,e^	7.8 ± 1.5^a,b,c,d^	0.7 ± 0.1^a,b,c^
(13) M^te^ Novo	91.7 ± 1.2^a,b,c,d,e^	7.6 ± 1.1^a,b,c,d^	0.7 ± 0.1^a,b,c^
(14) Montejuntos	92.7 ± 0.4^a,b,c,d^	6.7 ± 0.4^a,b,c^	0.5 ± 0.1^a,b^
(15) N. S^ra^ Guadalupe	91.4 ± 1.1^a,b,c,d,e^	7.9 ± 1.3^a,b,c,d^	0.7 ± 0.2^a,b,c^
(16) N. S^ra^ Machede	91.4 ± 1.5^a,b,c,d,e^	8.0 ± 1.4^a,b,c,d^	0.7 ± 0.2^a,b,c^
(17) Rosal de la Frontera	90.4 ± 1.7^b,c,d,e^	8.8 ± 1.6^b,c,d^	0.8 ± 0.1^a,b,c^
(18) S^to^ Aleixo da Restauração	91.1 ± 1.1^a,b,c,d,e^	8.1 ± 1.1^a,b,c,d^	0.8 ± 0.1^a,b,c^
(19) S. Miguel de Machede	92.7 ± 0.4^a,b,c,d^	6.5 ± 0.3^a,b,c^	0.7 ± 0.1^a,b,c^
(20) Serpa	93.8 ± 0.5^a^	5.8 ± 0.5^a^	0.5 ± 0.0^a^
(21) V^e^ Rocins	93.0 ± 0.7^a,b,c^	6.4 ± 0.7^a,b,c^	0.6 ± 0.1^a,b,c^
(22) Valverde	92.0 ± 1.6^a,b,c,d,e^	7.1 ± 1.6^a,b,c,d^	0.9 ± 0.0^c^
(23) V. N. S. Bento	90.0 ± 0.2^d,e^	9.1 ± 0.2^c,d^	0.8 ± 0.0^a,b,c^
(24) Villanueva del Fresno	93.1 ± 0.6^a,b^	6.4 ± 0.5^a,b,c^	0.5 ± 0.1^a^

Values of each determination represents mean ± SD (*n* = 9). Different letters following the values indicate significant differences (*p* < 0.05).

**Table 3:** Ca, Mg, Na, and K mineral content of *A. ponderosa* fruiting bodies and corresponding soil substrates from different sampling sites.

Sampling site	Sample	Minerals (mg/Kg dry weight)			
		Ca	K	Mg	Na
(1) Almendres	FB	757 ± 156^a^	26677 ± 915^a,b,c,d,e^	774 ± 130^a,b,c^	1408 ± 146^a^
	SS	106 ± 11^a,b^	363 ± 9^a,b^	286 ± 14^a,b,c,d^	70 ± 1^a,b,c,d^
(2) Azaruja	FB	139 ± 13^b^	4589 ± 103^a^	194 ± 10^a^	216 ± 135^f^
	SS	102 ± 12^a,b^	981 ± 37^g^	324 ± 29^a,b^	75 ± 4^a,b,h,i^
(3) Baleizão	FB	606 ± 61^a^	25758 ± 4728^a,b,c,d,e^	856 ± 107^a,b,c,d^	2249 ± 231^g,h^
	SS	162 ± 11^c,d^	1099 ± 24^h^	312 ± 38^a,b^	109 ± 4^k^
(4) Beja	FB	615 ± 1^a^	6090 ± 34^a,b^	734 ± 1^a,b,c^	1540 ± 17^a,g^
	SS	189 ± 14^d,e^	619 ± 19^d,e^	277 ± 33^a,b,c,d,e^	69 ± 2^a,b,c,d,e^
(5) Cabeça Gorda	FB	554 ± 21^a^	38652 ± 1607^c,d,e^	848 ± 159^a,b,c^	889 ± 38^a,b,c,d,e,f^
	SS	108 ± 6^a,b^	384 ± 16^a,b,c^	186 ± 8^e,f,g^	69 ± 5^a,b,c,d,e^
(6) Cabezas Rubias	FB	352 ± 2^c^	69565 ± 362^f^	1265 ± 1^c,d^	2645 ± 18^h^
	SS	118 ± 16^a,b,c^	443 ± 9^c^	277 ± 19^a,b,c,d,e^	62 ± 2^c,d,e,f^
(7) Évora	FB	605 ± 1^a^	34630 ± 186^c,d,e^	713 ± 1^a,b,c^	1241 ± 16^a,b^
	SS	91 ± 23^a^	651 ± 9^e^	171 ± 25^f,g^	52 ± 3^f,g^
(8) Évoramonte	FB	733 ± 15^a^	25148 ± 4556^a,b,c,d,e^	760 ± 101^a,b,c^	614 ± 131^b,c,d,e,f^
	SS	102 ± 8^a,b^	576 ± 16^d^	156 ± 11^g^	51 ± 2^g^
(9) Her^de^ da Mitra	FB	579 ± 111^a^	31852 ± 5364^a,b,c,d,e^	685 ± 89^a,b,c^	930 ± 58^a,b,c,d,e,f^
	SS	159 ± 8^c,d^	229 ± 9^j,k^	298 ± 29^a,b,c^	51 ± 3^g^
(10) Mértola	FB	370 ± 57^a,b^	18021 ± 1806^a,b,c^	658 ± 33^a,b,c^	484 ± 171^c,d,e,f^
	SS	112 ± 8^a,b,c^	731 ± 19^f^	193 ± 18^d,e,f,g^	82 ± 2^h,i,j^
(11) Mina S. Domingos	FB	582 ± 1^a^	33607 ± 173^b,c,d,e^	737 ± 1^a,b,c^	1097 ± 14^a,b,c,d^
	SS	143 ± 25^b,c,d^	256 ± 16^i,j^	365 ± 43^a^	59 ± 5^e,f,g^
(12) M^te^ da Borralha	FB	448 ± 33^a,b^	26099 ± 1618^a,b,c,d,e^	696 ± 29^a,b,c^	368 ± 70^e,f^
	SS	70 ± 14^a^	155 ± 9^l^	172 ± 23^f,g^	67 ± 5^b,c,d,e^
(13) M^te^ Novo	FB	436 ± 72^a,b^	25763 ± 1608^a,b,c,d,e^	562 ± 114^a,b^	379 ± 25^e,f^
	SS	148 ± 19^b,c,d^	603 ± 56^d,e^	217 ± 34^c,d,e,f,g^	53 ± 4^f,g^
(14) Montejuntos	FB	412 ± 50^a,b^	41285 ± 982^c,d,e^	742 ± 53^a,b,c^	1146 ± 304^a,b,c^
	SS	224 ± 16^e,f^	256 ± 16^i,j^	910 ± 25^j^	85 ± 2^i,j^
(15) N. S^ra^ Guadalupe	FB	408 ± 11^a,b^	34814 ± 1763^c,d,e^	930 ± 158^b,c,d^	551 ± 52^b,c,d,e,f^
	SS	148 ± 8^b,c,d^	656 ± 16^e^	255 ± 14^b,c,d,e,f^	73 ± 1^a,b,h^
(16) N. S^ra^ de Machede	FB	632 ± 111^a^	24717 ± 1881^a,b,c,d,e^	843 ± 120^a,b,c^	2185 ± 438^g,h^
	SS	143 ± 3^b,c,d^	912 ± 48^g^	804 ± 48^i^	80 ± 2^a,h,i,j^
(17) Rosal de la Frontera	FB	584 ± 25^a^	51996 ± 2660^e,f^	1540 ± 77^d^	964 ± 453^a,b,c,d,e^
	SS	83 ± 9^a^	160 ± 16^k,l^	365 ± 11^a^	80 ± 1^a,h,i,j^
(18) S^to^ Aleixo da Restauração	FB	366 ± 1^a^	18586 ± 106^d,e,f^	481 ± 1^a,b,c^	1098 ± 103^a,b,c,d^
	SS	79 ± 8^a^	773 ± 24^f^	477 ± 14^h^	70 ± 2^a,b,c^
(19) S. Miguel de Machede	FB	649 ± 74^a^	45011 ± 1152^c,d,e,f^	549 ± 140^a,b^	897 ± 11^a,b,c,d,e,f^
	SS	338 ± 35^a,b,c^	315 ± 9^k,l^	1466 ± 68^a,b,c,d,e^	158 ± 5^c,d,e,f,g^
(20) Serpa	FB	698 ± 148^a,b^	47715 ± 5220^a,b,c^	736 ± 86^a,b^	1078 ± 174^a,b,c,d,e^
	SS	119 ± 16^g^	171 ± 9^a,i^	272 ± 15^k^	60 ± 5^l^
(21) V^e^ Rocins	FB	448 ± 269^a,b^	21788 ± 1237^a,b,c,d^	418 ± 203^a,b^	419 ± 212^d,e,f^
	SS	113 ± 8^a,b,c^	320 ± 16^a,i^	284 ± 14^a,b,c,d^	86 ± 2^j^
(22) Valverde	FB	521 ± 146^a,b^	27256 ± 2180^a,b,c,d,e^	586 ± 35^a,b,c^	895 ± 65^a,b,c,d,e,f^
	SS	93 ± 23^a^	405 ± 24^b,c^	265 ± 41^b,c,d,e,f^	111 ± 3^k^
(23) V. N. S. Bento	FB	599 ± 1^a^	6228 ± 30^a,b^	657 ± 1^a,b,c^	1467 ± 15^a^
	SS	233 ± 11^e,f^	192 ± 16^j,k,l^	536 ± 37^h^	60 ± 2^d,e,f,g^
(24) Villanueva del Fresno	FB	459 ± 13^a,b^	25713 ± 5847^a,b,c,d,e^	738 ± 49^a,b,c^	1451 ± 147^a^
	SS	240 ± 26^f^	368 ± 16^a,b^	267 ± 27^b,c,d,e^	78 ± 4^a,h,i,j^

Total	FB	523 ± 143	29648 ± 14908	738 ± 261	1092 ± 620
	SS	143 ± 63	484 ± 273	381 ± 296	75 ± 24

FB, fruiting bodies; SS, soil substrate. Mean values (*n* = 9)  ± SD. Different letters for each element indicate significant differences with the confidence level of *p* < 0.05 (ANOVA, Tukey's test).

**Table 4 tab4:** Al, Cu, Fe, and P mineral content of *A. ponderosa* fruiting bodies and corresponding soil substrates from different sampling sites.

Sampling site	Sample	Minerals (mg/Kg dry weight)			
		Al	Cu	Fe	P
(1) Almendres	FB	349 ± 124^b,c,d,e,f^	80 ± 1^a,b^	66 ± 14^a,b,c^	319 ± 37^a,b,c,d,e^
	SS	277 ± 8^a,b^	1 ± 0^a^	1547 ± 92^a^	72 ± 2^a,b^
(2) Azaruja	FB	58 ± 8^a^	30 ± 2^a^	18 ± 4^a^	44 ± 18^h,i^
	SS	243 ± 30^a^	2 ± 0^c^	2240 ± 160^b^	74 ± 4^a,b^
(3) Baleizão	FB	357 ± 29^b,c,d,e,f^	356 ± 37^a,b,c^	27 ± 8^a^	525 ± 62^j,k^
	SS	397 ± 4^c,d,e^	7 ± 0^k^	3093 ± 92^d,e,f^	156 ± 4^g^
(4) Beja	FB	449 ± 13^d,e,f,g^	373 ± 1^b,c^	227 ± 1^f,g^	212 ± 1^d,e,f,g^
	SS	254 ± 15^a^	9 ± 1^l^	4107 ± 92^i,j^	133 ± 8^f^
(5) Cabeça Gorda	FB	151 ± 25^a,b^	161 ± 10^a,b^	51 ± 12^a,b,c^	447 ± 2^a,j^
	SS	196 ± 4^k^	1 ± 0^a^	5067 ± 244^k^	130 ± 3^e,f^
(6) Cabezas Rubias	FB	932 ± 77^h^	140 ± 1^a,b^	193 ± 1^e,f^	73 ± 1^g,h,i^
	SS	375 ± 8^c,d^	5 ± 0^g^	3520 ± 160^e,f,g,h^	93 ± 4^c^
(7) Évora	FB	208 ± 11^a,b,c,d^	198 ± 1^a,b^	292 ± 1^g,h^	201 ± 1^d,e,f,g,h^
	SS	368 ± 18^c^	2 ± 0^b,c^	1653 ± 92^a^	82 ± 5^b,c^
(8) Évoramonte	FB	423 ± 49^c,d,e,f,g^	124 ± 20^a,b^	62 ± 7^a,b,c^	469 ± 83^a,j^
	SS	311 ± 6^b^	2 ± 0^c,d^	2400 ± 160^b,c^	94 ± 1^c^
(9) Her^de^ da Mitra	FB	373 ± 26^b,c,d,e,f^	193 ± 19^a,b^	35 ± 10^a,b^	277 ± 90^b,c,d,e,f^
	SS	157 ± 6^l^	10 ± 0^m^	4747 ± 92^k^	115 ± 3^d,e^
(10) Mértola	FB	333 ± 36^b,c,d,e^	43 ± 15^a,b^	22 ± 3^a^	61 ± 21^g,h,i^
	SS	160 ± 8^l^	5 ± 0^f,g^	4853 ± 244^k^	119 ± 2^d,e,f^
(11) Mina S. Domingos	FB	299 ± 14^a,b,c,d,e^	334 ± 1^a,b,c^	62 ± 1^a,b,c^	149 ± 1^f,g,h,i^
	SS	417 ± 6^e,f^	3 ± 0^d,e^	3573 ± 92^f,g,h^	74 ± 2^a,b^
(12) M^te^ da Borralha	FB	209 ± 42^a,b,c,d^	232 ± 63^a,b^	41 ± 38^a,b^	417 ± 65^a,b,c,j^
	SS	159 ± 6^l^	2 ± 0^c^	3840 ± 160^h,i^	75 ± 5^a,b^
(13) M^te^ Novo	FB	423 ± 59^c,d,e,f,g^	121 ± 16^a,b^	29.3 ± 1^a^	316 ± 76^a,b,c,d,e,f^
	SS	300 ± 15^b^	3 ± 0^c,d^	1653 ± 244^a^	84 ± 4^b,c^
(14) Montejuntos	FB	545 ± 99^e,f,g^	170 ± 21^a,b^	62 ± 10^a,b,c^	442 ± 2^a,b,j^
	SS	813 ± 11^j^	7 ± 0^j,k^	8099 ± 251^l^	294 ± 11^i^
(15) N. S^ra^ Guadalupe	FB	209 ± 73^a,b,c,d^	264 ± 23^a,b,c^	110 ± 27^b,c,d^	344 ± 74^a,b,c,d^
	SS	200 ± 6^k^	8 ± 0^l^	4960 ± 160^k^	131 ± 5^e,f^
(16) N. S^ra^ de Machede	FB	313 ± 47^a,b,c,d,e^	148 ± 26^a,b^	48 ± 13^a,b,c^	542 ± 48^j,k^
	SS	632 ± 19^i^	4 ± 0^e,f^	3738 ± 258^g,h,i^	78 ± 1^a,b,c^
(17) Rosal de la Frontera	FB	659 ± 36^g^	584 ± 51^c^	119 ± 97^c,d,e^	641 ± 98^k^
	SS	381 ± 4^c,d^	9 ± 0^l^	3360 ± 160^e,f,g,h^	154 ± 4^g^
(18) S^to^ Aleixo da Restauração	FB	65 ± 12^d,e,f,g^	129 ± 1^a,b^	51 ± 1^a,b,c^	34 ± 1^a,j^
	SS	449 ± 16^f,g^	6 ± 0^g,h,i^	2827 ± 92^c,d^	185 ± 1^h^
(19) S. Miguel de Machede	FB	610 ± 183^f,g^	207 ± 39^a,b^	311 ± 10^h^	270 ± 60^c,d,e,f^
	SS	497 ± 6^c,d^	5 ± 0^g,h^	9040 ± 139^d,e,f,g^	111 ± 2^h^
(20) Serpa	FB	453 ± 66^a^	143 ± 14^a,b^	43 ± 18^a,b,c^	460 ± 16^i^
	SS	378 ± 4^h^	5 ± 0^f,g^	3253 ± 92^m^	179 ± 7^d^
(21) V^e^ Rocins	FB	181 ± 24^a,b,c^	84 ± 7^a,b^	36 ± 24^a,b^	165 ± 85^e,f,g,h,i^
	SS	391 ± 9^c,d,e^	9 ± 0^l^	3040 ± 160^d,e^	75 ± 5^a,b^
(22) Valverde	FB	183 ± 54^a,b,c^	62 ± 3^a,b^	57 ± 21^a,b,c^	152 ± 34^f,g,h,i^
	SS	453 ± 6^g^	1 ± 0^a,b^	1440 ± 160^a^	65 ± 2^a^
(23) V. N. S. Bento	FB	303 ± 7^a,b,c,d,e^	172 ± 1^a,b^	182 ± 1^d,e,f^	197 ± 1^d,e,f,g,h,i^
	SS	409 ± 6^d,e^	6 ± 0^h,i,j^	4587 ± 244^j,k^	116 ± 5^d,e^
(24) Villanueva del Fresno	FB	602 ± 66^f,g^	100 ± 9^a,b^	53 ± 17^a,b,c^	310 ± 83^a,b,c,d,e,f^
	SS	494 ± 4^h^	6 ± 0^i,j^	2347 ± 92^b,c^	179 ± 12^h^

Total	FB	362 ± 204	185 ± 125	92 ± 85	294 ± 171
	SS	363 ± 155	5 ± 3	3708 ± 1870	120 ± 53

FB, fruiting bodies; SS, soil substrate. Mean values (*n* = 9)  ± SD. Different letters for each element indicate significant differences with the confidence level of *p* < 0.05 (ANOVA, Tukey's test).

**Table 5 tab5:** Ag, Ba, Cd, and Cr mineral content of *A. ponderosa* fruiting bodies and corresponding soil substrates from different sampling sites.

Sampling site	Sample	Minerals (mg/kg dry weight)			
		Ag	Ba	Cd	Cr
(1) Almendres	FB	2.6 ± 0.5^a,b,c,d,e^	2.9 ± 1.1^a^	2.2 ± 0.8^a^	1.3 ± 0.6^a,b,c^
	SS	0.1 ± 0.0^a^	2.0 ± 0.3^a,b^	0.3 ± 0.0^a,b^	1.0 ± 0.2^a^
(2) Azaruja	FB	6.6 ± 2.2^f^	0.9 ± 0.2^d,e,f,g^	0.3 ± 0.0^b^	0.6 ± 0.1^c,d,e^
	SS	0.1 ± 0.0^a,b,c,d^	1.6 ± 0.2^a^	0.3 ± 0.1^a^	0.9 ± 0.1^a^
(3) Baleizão	FB	5.1 ± 1.8^a,b,c,d^	0.6 ± 0.3^d,e,f,g^	1.1 ± 0.3^b,c,d,e^	1.0 ± 0.2^d,e^
	SS	0.1 ± 0.0^a,b,c,d^	5.3 ± 0.9^i^	0.2 ± 0.0^e^	2.4 ± 0.1^b,c,d^
(4) Beja	FB	1.1 ± 0.2^a,b^	0.8 ± 0.0^b,c,d,e,f,g^	0.4 ± 0.0^b,c,d,e^	0.8 ± 0.0^a^
	SS	0.1 ± 0.0^a,b,c,d,e,f^	2.6 ± 0.7^a,b,c,d^	0.1 ± 0.0^f^	4.4 ± 0.2^i,j^
(5) Cabeça Gorda	FB	2.1 ± 0.7^a,b,c^	0.3 ± 0.0^d,e,f,g^	0.7 ± 0.1^c,d,e^	0.8 ± 0.0^b,c,d,e^
	SS	0.2 ± 0.0^b,c,d,e,f^	2.3 ± 0.1^a,b,c,d^	0.2 ± 0.0^c,d,e^	4.2 ± 0.0^g,h,i,j^
(6) Cabezas Rubias	FB	1.5 ± 0.2^a,b,c^	2.0 ± 0.3^d,e,f,g^	0.8 ± 0.1^c,d,e^	0.9 ± 0.1^f^
	SS	0.2 ± 0.0^c,d,e,f,g^	2.6 ± 0.4^a,b,c,d^	0.1 ± 0.0^e,f^	3.1 ± 0.5^c,d,e,f,g,h^
(7) Évora	FB	0.5 ± 0.3^a,b,c,d^	0.8 ± 0.4^f,g^	0.4 ± 0.3^b,c,d,e^	0.9 ± 0.5^f^
	SS	0.2 ± 0.0^g,h,i^	4.3 ± 1.1^f,g,h,i^	0.1 ± 0.0^e,f^	2.8 ± 0.5^c,d,e,f^
(8) Évoramonte	FB	1.0 ± 0.1^d,e,f^	0.9 ± 0.1^b,c,d,e^	1.2 ± 0.2^b,c,d,e^	2.9 ± 0.1^b,c,d,e^
	SS	0.1 ± 0.0^a,b,c,d,e,f^	2.8 ± 0.1^a,b,c,d,e^	0.1 ± 0.0^e,f^	2.6 ± 0.0^c,d,e,f^
(9) Her^de^ da Mitra	FB	0.7 ± 0.1^a,b,c,d^	1.2 ± 0.1^g^	1.0 ± 0.0^b,c,d,e^	1.8 ± 0.1^b,c,d,e^
	SS	0.2 ± 0.0^e,f,g,h^	2.3 ± 0.1^a,b,c^	0.1 ± 0.0^e,f^	3.8 ± 0.1^f,g,h,i,j^
(10) Mértola	FB	4.3 ± 0.8^a,b^	1.5 ± 0.2^b,c,d,e,f^	1.0 ± 0.3^b,c,d^	0.9 ± 0.3^d,e^
	SS	0.1 ± 0.0^a,b^	3.0 ± 0.4^b,c,d,e,f^	0.1 ± 0.0^e,f^	3.0 ± 0.2^c,d,e,f,g^
(11) Mina S. Domingos	FB	0.6 ± 0.1^a,b,c^	0.6 ± 0.0^c,d,e,f,g^	1.4 ± 0.2^d,e^	0.7 ± 0.1^a,b,c,d,e^
	SS	0.3 ± 0.0^i^	1.9 ± 0.0^a,b^	0.1 ± 0.0^e,f^	5.1 ± 0.7^j,k^
(12) M^te^ da Borralha	FB	1.5 ± 0.1^a,b,c^	0.9 ± 0.1^f,g^	1.1 ± 0.2^b,c^	0.9 ± 0.1^c,d,e^
	SS	0.2 ± 0.0^d,e,f,g^	2.3 ± 0.0^a,b,c,d^	0.1 ± 0.0^e,f^	3.7 ± 0.0^d,e,f,g,h,i^
(13) M^te^ Novo	FB	0.6 ± 0.1^a^	1.3 ± 0.2^e,f,g^	0.6 ± 0.1^b,c,d,e^	0.5 ± 0.1^e^
	SS	0.1 ± 0.0^a,b,c^	3.4 ± 0.2^c,d,e,f,g^	0.2 ± 0.0^b,c,d,e^	2.6 ± 0.2^c,d,e,f^
(14) Montejuntos	FB	1.5 ± 0.2^a,b,c^	1.1 ± 0.1^b,c,d^	1.3 ± 0.1^b,c,d,e^	1.0 ± 0.1^a,b,c,d^
	SS	0.2 ± 0.1^h,i^	3.6 ± 0.3^d,e,f,g,h^	0.1 ± 0.0^e,f^	5.7 ± 0.2^k^
(15) N. S^ra^ Guadalupe	FB	2.5 ± 0.3^e,f^	0.7 ± 0.2^d,e,f,g^	0.9 ± 0.2^b,c,d,e^	0.5 ± 0.1^a,b,c,d,e^
	SS	0.2 ± 0.0^b,c,d,e,f,g^	2.4 ± 0.1^a,b,c,d^	0.2 ± 0.0^c,d,e^	4.3 ± 0.1^h,i,j^
(16) N. S^ra^ Machede	FB	0.4 ± 0.2^a,b,c,d,e^	0.6 ± 0.1^b,c,d,e,f,g^	0.7 ± 0.1^f^	0.4 ± 0.1^b,c,d,e^
	SS	0.4 ± 0.0^j^	4.0 ± 0.2^e,f,g,h,i^	0.1 ± 0.0^e,f^	3.8 ± 0.3^e,f,g,h,i,j^
(17) Rosal de la Frontera	FB	1.6 ± 0.3^c,d,e^	1.5 ± 0.1^b,c,d,e,f^	1.0 ± 0.1^b,c^	1.2 ± 0.0^a^
	SS	0.2 ± 0.0^c,d,e,f,g^	2.3 ± 0.1^a,b,c,d^	0.1 ± 0.0^e,f^	2.9 ± 0.2^c,d,e,f^
(18) S^to^ Aleixo da Restauração	FB	1.3 ± 0.3^a,b,c^	1.1 ± 0.1^d,e,f,g^	0.7 ± 0.1^b,c^	0.9 ± 0.0^b,c,d,e^
	SS	0.2 ± 0.0^e,f,g,h^	2.8 ± 0.2^a,b,c,d,e^	0.3 ± 0.0^a,b,c,d^	2.8 ± 0.9^c,d,e,f^
(19) S. Miguel de Machede	FB	2.6 ± 0.3^b,c,d,e^	1.3 ± 0.3^b,c,d,e,f,g^	3.9 ± 0.5^b,c,d,e^	0.9 ± 0.1^a,b^
	SS	0.2 ± 0.0^e,f,g^	3.4 ± 0.1^c,d,e,f,g^	0.1 ± 0.0^e,f^	2.3 ± 0.2^b,c^
(20) Serpa	FB	1.7 ± 0.3^a,b,c^	0.4 ± 0.1^a,b,c^	0.4 ± 0.1^b,c,d,e^	0.6 ± 0.1^b,c,d,e^
	SS	0.2 ± 0.0^f,g,h^	2.4 ± 0.3^a,b,c,d^	0.0 ± 0.0^f^	7.9 ± 0.4^l^
(21) V^e^ Rocins	FB	3.3 ± 2.2^a^	1.3 ± 0.5^d,e,f,g^	0.5 ± 0.2^b,c^	1.8 ± 0.6^b,c,d,e^
	SS	0.1 ± 0.0^a,b,c,d,e^	4.8 ± 0.2^h,i^	0.2 ± 0.0^d,e^	2.5 ± 0.1^b,c,d,e^
(22) Valverde	FB	1.8 ± 0.6^a,b^	0.5 ± 0.0^d,e,f,g^	1.0 ± 0.1^e^	2.9 ± 0.1^b,c,d,e^
	SS	0.2 ± 0.0^c,d,e,f,g^	1.7 ± 0.3^a,b^	0.2 ± 0.0^b,c,d,e^	1.2 ± 0.1^a,b^
(23) V. N. S. Bento	FB	0.4 ± 0.3^a,b,c^	2.1 ± 0.2^c,d,e,f,g^	0.7 ± 0.1^b,c,d,e^	2.8 ± 0.4^b,c,d,e^
	SS	0.1 ± 0.0^a,b,c,d,e,f^	4.5 ± 0.4^g,h,i^	0.3 ± 0.1^a,b,c^	1.8 ± 1.3^a,b,c^
(24) Villanueva del Fresno	FB	3.0 ± 0.3^a^	1.1 ± 0.2^a,b^	0.8 ± 0.3^b,c,d,e^	1.5 ± 0.4^f^
	SS	0.1 ± 0.0^a,b,c,d,e^	11.3 ± 1.0^j^	0.3 ± 0.0^a,b,c^	4.9 ± 0.6^i,j,k^

Total	FB	2.0 ± 1.6	1.1 ± 0.6	1.0 ± 0.7	1.2 ± 0.7
	SS	0.2 ± 0.1	3.3 ± 2.0	0.2 ± 0.1	3.3 ± 1.6

FB, fruiting bodies; SS, soil substrate. Mean values (*n* = 9)  ±  SD. Different letters for each element indicate significant differences with the confidence level of *p* < 0.05 (ANOVA, Tukey's test).

**Table 6:** Mn, Pb, and Zn mineral content of* A. ponderosa* fruiting bodies and corresponding soil substrates from different sampling sites.

Sampling site	Sample	Minerals (mg/kg dry weight)		
		Mn	Pb	Zn
(1) Almendres	FB	28 ± 6^a,b,c,d^	0.7 ± 0.5^a,b^	59 ± 1^a,b,c,d^
	SS	22 ± 1^a^	4.0 ± 0.4^a^	4 ± 0^a,b^
(2) Azaruja	FB	6 ± 1^a^	0.4 ± 0.1^a^	16 ± 2^a^
	SS	131 ± 9^a,b^	7.0 ± 1.0^a,b,c,d,e^	5 ± 1^a,b,c^
(3) Baleizão	FB	91 ± 14^g,h^	1.7 ± 0.5^c,d,e,f^	97 ± 2^c,d,e^
	SS	893 ± 17^f,g^	9.2 ± 1.1^d,e,f^	9 ± 0^d^
(4) Beja	FB	84 ± 1^f,g,h^	2.8 ± 0.1^a,b,c,d^	104 ± 1^d,e^
	SS	507 ± 45^c^	6.9 ± 0.6^a,b,c,d,e^	13 ± 0^g^
(5) Cabeça Gorda	FB	66 ± 6^c,d,e,f,g,h^	1.9 ± 0.2^c,d,e,f^	81 ± 1^b,c,d,e^
	SS	695 ± 62^d,e^	4.5 ± 0.2^a,b^	10 ± 0^d^
(6) Cabezas Rubias	FB	81 ± 1^e,f,g,h^	5.1 ± 0.8^h,i^	132 ± 1^e^
	SS	735 ± 30^d,e,f^	16.5 ± 0.6^g^	11 ± 1^d,e^
(7) Évora	FB	59 ± 1^b,c,d,e,f,g,h^	1.9 ± 0.9^i^	75 ± 1^b,c,d,e^
	SS	175 ± 3^a,b^	10.9 ± 0.2^f^	5 ± 1^b,c^
(8) Évoramonte	FB	79 ± 11^e,f,g,h^	4.2 ± 0.2^g,h,i^	59 ± 7^a,b,c,d^
	SS	212 ± 12^b^	7.4 ± 0.3^a,b,c,d,e,f^	6 ± 0^c^
(9) Her^de^ da Mitra	FB	47 ± 9^a,b,c,d,e,f,g^	1.4 ± 0.2^b,c,d,e,f^	61 ± 2^a,b,c,d^
	SS	725 ± 17^d,e^	5.4 ± 0.3^a,b,c^	11 ± 0^e,f^
(10) Mértola	FB	29 ± 6^a,b,c,d^	3.7 ± 0.9^a,b,c,d,e^	52 ± 8^a,b,c,d^
	SS	725 ± 17^d,e^	8.0 ± 0.7^b,c,d,e,f^	15 ± 1^h^
(11) Mina S. Domingos	FB	104 ± 1^h^	3.1 ± 0.7^e,f,g,h^	91 ± 1^c,d,e^
	SS	290 ± 6^b^	10.3 ± 0.4^ e,f^	4 ± 1^a^
(12) M^te^ da Borralha	FB	39 ± 16^a,b,c,d,e,f^	2.2 ± 0.3^a,b,c^	62 ± 4^a,b,c,d^
	SS	199 ± 9^b^	5.4 ± 0.3^a,b,c^	10 ± 1^d,e^
(13) M^te^ Novo	FB	34 ± 4^a,b,c,d^	1.6 ± 0.3^a^	52 ± 4^a,b,c,d^
	SS	139 ± 3^a,b^	8.2 ± 0.7^c,d,e,f^	5 ± 0^a,b,c^
(14) Montejuntos	FB	49 ± 9^a,b,c,d,e,f,g^	3.0 ± 0.1^e,f,g,h^	60 ± 3^a,b,c,d^
	SS	649 ± 27^c,d,e^	10.2 ± 1.5^e,f^	10 ± 0^d,e^
(15) N. S^ra^ Guadalupe	FB	38 ± 5^a,b,c,d,e^	2.1 ± 0.3^a,b,c,d,e,f^	63 ± 10^a,b,c,d^
	SS	794 ± 232^e,f^	4.5 ± 0.2^a,b^	12 ± 0^f,g^
(16) N. S^ra^ Machede	FB	50 ± 13^a,b,c,d,e,f,g^	0.4 ± 0.1^a,b,c^	70 ± 2^a,b,c,d^
	SS	123 ± 4^a,b^	8.4 ± 0.1^c,d,e,f^	9 ± 0^d^
(17) Rosal de la Frontera	FB	90 ± 56^g,h^	3.0 ± 0.1^c,d,e,f,g^	95 ± 6^c,d,e^
	SS	725 ± 17^d,e^	16.5 ± 0.6^g^	6 ± 0^b,c^
(18) S^to^ Aleixo da Restauração	FB	47 ± 15^c,d,e,f,g,h^	3.9 ± 0.3^i^	47 ± 1^a,b,c,d^
	SS	193 ± 7^b^	6.4 ± 0.9^a,b,c,d^	11 ± 0^d,e^
(19) S. Miguel de Machede	FB	91 ± 1^g,h^	1.4 ± 0.1^f,g,h^	58 ± 8^a,b,c,d^
	SS	576 ± 17^g^	5.8 ± 0.2^a,b,c,d^	9 ± 0^d^
(20) Serpa	FB	69 ± 19^a,b,c,d,e,f,g^	1.0 ± 0.2^d,e,f,g,h^	61 ± 9^a,b,c,d^
	SS	1002 ± 30^c,d^	19.5 ± 3.7^g^	30 ± 1^j^
(21) V^e^ Rocins	FB	15 ± 10^a,b^	2.3 ± 0.8^b,c,d,e,f^	31 ± 2^a,b^
	SS	893 ± 17^f,g^	9.2 ± 1.1^d,e,f^	5 ± 1^a,b,c^
(22) Valverde	FB	26 ± 7^a,b,c^	5.1 ± 0.2^f,g,h^	42 ± 2^a,b,c^
	SS	21 ± 1^a^	4.4 ± 0.4^a,b^	5 ± 0^a,b,c^
(23) V. N. S. Bento	FB	72 ± 1^d,e,f,g,h^	1.6 ± 0.1^h,i^	86 ± 1^b,c,d,e^
	SS	745 ± 45^e,f^	8.4 ± 3.0^c,d,e,f^	27 ± 1^i^
(24) Villanueva del Fresno	FB	39 ± 6^a,b,c,d,e,f^	3.1 ± 0.6^a,b,c,d,e^	68 ± 4^a,b,c,d^
	SS	1518 ± 45^h^	7.4 ± 0.5^a,b,c,d,e,f^	5 ± 1^a,b,c^

Total	FB	56 ± 27	2.4 ± 1.3	68 ± 25
	SS	529 ± 378	8.5 ± 4.0	10 ± 6

FB, fruiting bodies; SS, soil substrate. Mean values (*n* = 9)  ± SD. Different letters for each element indicate significant differences with the confidence level of *p* < 0.05 (ANOVA, Tukey's test).

**Table 7:** Bioconcentration factor (BCF) values for *A. ponderosa* fruiting bodies from different sampling sites.

Sampling site	Al	Ca	Cu	Fe	K	Mg	Na	P
(1) Almendres	1.3 ± 0.4	7.1 ± 0.7	123 ± 2	0.04 ± 0.01	74 ± 1	2.7 ± 0.3	20 ± 2	4.4 ± 0.4
(2) Azaruja	0.2 ± 0.0	1.4 ± 0.0	16 ± 1	0.01 ± 0.00	5 ± 1	0.6 ± 0.0	3 ± 2	0.6 ± 0.2
(3) Baleizão	0.9 ± 0.1	3.7 ± 0.1	48 ± 3	0.01 ± 0.00	23 ± 4	2.7 ± 0.0	21 ± 1	3.4 ± 0.3
(4) Beja	1.8 ± 0.1	3.3 ± 0.2	43 ± 2	0.06 ± 0.00	10 ± 0	2.7 ± 0.3	22 ± 0	1.6 ± 0.1
(5) Cabeça Gorda	0.8 ± 0.1	5.1 ± 0.1	248 ± 16	0.01 ± 0.00	101 ± 0	4.5 ± 0.7	13 ± 0	3.4 ± 0.1
(6) Cabezas Rubias	2.5 ± 0.2	3.0 ± 0.4	28 ± 1	0.05 ± 0.00	157 ± 2	4.6 ± 0.3	43 ± 1	0.8 ± 0.0
(7) Évora	0.6 ± 0.0	7.0 ± 1.8	122 ± 19	0.18 ± 0.01	53 ± 0	4.2 ± 0.6	24 ± 1	2.5 ± 0.1
(8) Évoramonte	1.4 ± 0.1	7.2 ± 0.4	52 ± 1	0.03 ± 0.00	44 ± 7	4.9 ± 0.3	12 ± 2	5.0 ± 0.8
(9) Her^de^ da Mitra	2.4 ± 0.1	3.6 ± 0.5	20 ± 1	0.01 ± 0.00	139 ± 18	2.3 ± 0.1	18 ± 0	2.4 ± 0.7
(10) Mértola	2.1 ± 0.1	3.3 ± 0.3	9 ± 3	0.00 ± 0.00	25 ± 2	3.4 ± 0.2	6 ± 2	0.5 ± 0.2
(11) Mina S. Domingos	0.7 ± 0.0	4.2 ± 0.7	114 ± 10	0.02 ± 0.00	132 ± 8	2.0 ± 0.2	19 ± 1	2.0 ± 0.0
(12) M^te^ da Borralha	1.3 ± 0.2	6.5 ± 0.9	119 ± 32	0.01 ± 0.01	168 ± 1	4.1 ± 0.4	5 ± 1	5.5 ± 0.5
(13) M^te^ Novo	1.4 ± 0.1	2.9 ± 0.1	48 ± 2	0.02 ± 0.00	43 ± 1	2.6 ± 0.1	7 ± 0	3.7 ± 0.7
(14) Montejuntos	0.7 ± 0.1	1.8 ± 0.1	25 ± 1	0.01 ± 0.00	162 ± 6	0.8 ± 0.0	13 ± 3	1.5 ± 0.1
(15) N. S^ra^ Guadalupe	1.0 ± 0.3	2.8 ± 0.1	31 ± 1	0.02 ± 0.00	53 ± 10	3.6 ± 0.4	8 ± 1	2.6 ± 0.5
(16) N. S^ra^ Machede	0.5 ± 0.1	4.4 ± 0.7	39 ± 7	0.01 ± 0.00	27 ± 1	1.1 ± 0.1	27 ± 5	6.9 ± 0.5
(17) Rosal de la Frontera	1.7 ± 0.1	7.1 ± 0.5	68 ± 4	0.03 ± 0.03	326 ± 16	4.2 ± 0.1	12 ± 6	4.2 ± 0.5
(18) S^to^ Aleixo da Restauração	0.1 ± 0.0	4.7 ± 0.5	23 ± 1	0.02 ± 0.00	24 ± 1	1.0 ± 0.0	16 ± 1	0.2 ± 0.0
(19) S. Miguel de Machede	1.2 ± 0.4	1.9 ± 0.0	38 ± 4	0.03 ± 0.00	142 ± 33	0.4 ± 0.1	6 ± 0	2.4 ± 0.5
(20) Serpa	1.2 ± 0.2	5.8 ± 0.5	31 ± 1	0.01 ± 0.01	278 ± 16	2.7 ± 0.2	18 ± 1	2.6 ± 0.0
(21) V^e^ Rocins	0.5 ± 0.1	3.9 ± 2.1	10 ± 1	0.01 ± 0.01	67 ± 34	1.5 ± 0.6	5 ± 2	2.2 ± 1.0
(22) Valverde	0.4 ± 0.1	5.6 ± 0.2	83 ± 24	0.04 ± 0.01	65 ± 50	2.2 ± 0.2	8 ± 0	2.3 ± 0.5
(23) V. N. S. Bento	0.7 ± 0.0	2.6 ± 0.1	28 ± 1	0.04 ± 0.00	33 ± 3	1.2 ± 0.1	24 ± 1	1.7 ± 0.1
(24) Villanueva del Fresno	1.2 ± 0.1	1.9 ± 0.2	16 ± 1	0.02 ± 0.01	70 ± 13	2.8 ± 0.1	19 ± 1	1.7 ± 0.4

Values of each determination represents mean ± SD (*n* = 3).

**Table 8:** Bioconcentration factor (BCF) values for *A. ponderosa* fruiting bodies from different sampling sites.

Sampling site	Ag	Ba	Cd	Cr	Mn	Pb	Zn
(1) Almendres	38 ± 4	1.41 ± 0.33	7 ± 2	1.30 ± 0.39	1.27 ± 0.22	0.15 ± 0.10	13 ± 0
(2) Azaruja	72 ± 8	0.57 ± 0.04	1 ± 0	0.68 ± 0.03	0.05 ± 0.00	0.06 ± 0.00	3 ± 0
(3) Baleizão	55 ± 8	0.12 ± 0.04	7 ± 0	0.42 ± 0.07	0.10 ± 0.01	0.19 ± 0.03	10 ± 0
(4) Beja	8 ± 1	0.33 ± 0.08	15 ± 1	0.19 ± 0.00	0.17 ± 0.01	0.41 ± 0.02	8 ± 0
(5) Cabeça Gorda	14 ± 4	0.14 ± 0.01	4 ± 0	0.18 ± 0.01	0.09 ± 0.00	0.41 ± 0.03	8 ± 0
(6) Cabezas Rubias	10 ± 1	0.77 ± 0.01	8 ± 1	0.29 ± 0.00	0.11 ± 0.00	0.31 ± 0.04	13 ± 1
(7) Évora	2 ± 1	0.19 ± 0.05	4 ± 2	0.30 ± 0.13	0.34 ± 0.00	0.17 ± 0.08	15 ± 2
(8) Évoramonte	7 ± 0	0.32 ± 0.02	9 ± 1	1.14 ± 0.02	0.37 ± 0.03	0.56 ± 0.00	10 ± 1
(9) Her^de^ da Mitra	4 ± 0	0.53 ± 0.02	11 ± 2	0.47 ± 0.00	0.06 ± 0.01	0.26 ± 0.03	5 ± 0
(10) Mértola	61 ± 2	0.50 ± 0.01	8 ± 1	0.32 ± 0.07	0.04 ± 0.01	0.46 ± 0.07	3 ± 0
(11) Mina S. Domingos	2 ± 0	0.30 ± 0.01	13 ± 1	0.13 ± 0.00	0.36 ± 0.00	0.30 ± 0.06	25 ± 3
(12) M^te^ da Borralha	9 ± 0	0.40 ± 0.04	12 ± 1	0.24 ± 0.02	0.19 ± 0.07	0.41 ± 0.03	6 ± 0
(13) M^te^ Novo	6 ± 0	0.39 ± 0.05	3 ± 0	0.20 ± 0.02	0.24 ± 0.02	0.20 ± 0.02	10 ± 0
(14) Montejuntos	6 ± 1	0.29 ± 0.00	10 ± 2	0.18 ± 0.01	0.08 ± 0.01	0.30 ± 0.04	6 ± 0
(15) N. S^ra^ Guadalupe	16 ± 1	0.31 ± 0.08	5 ± 1	0.12 ± 0.01	0.05 ± 0.01	0.48 ± 0.06	5 ± 1
(16) N. S^ra^ Machede	1 ± 1	0.14 ± 0.02	8 ± 1	0.10 ± 0.00	0.40 ± 0.09	0.05 ± 0.02	7 ± 0
(17) Rosal de la Frontera	11 ± 2	0.65 ± 0.01	9 ± 1	0.42 ± 0.02	0.12 ± 0.07	0.18 ± 0.00	16 ± 1
(18) S^to^ Aleixo da Restauração	7 ± 0	0.39 ± 0.00	3 ± 0	0.36 ± 0.10	0.24 ± 0.07	0.62 ± 0.04	4 ± 0
(19) S. Miguel de Machede	16 ± 1	0.38 ± 0.08	38 ± 1	0.41 ± 0.03	0.16 ± 0.00	0.23 ± 0.01	6 ± 1
(20) Serpa	9 ± 0	0.18 ± 0.02	14 ± 3	0.07 ± 0.01	0.07 ± 0.02	0.05 ± 0.00	2 ± 0
(21) V^e^ Rocins	35 ± 21	0.27 ± 0.10	3 ± 1	0.71 ± 0.24	0.02 ± 0.01	0.25 ± 0.06	6 ± 0
(22) Valverde	12 ± 2	0.31 ± 0.04	5 ± 0	2.38 ± 0.03	1.23 ± 0.28	1.16 ± 0.05	9 ± 0
(23) V. N. S. Bento	4 ± 2	0.46 ± 0.01	2 ± 0	2.46 ± 2.01	0.10 ± 0.00	0.21 ± 0.07	3 ± 0
(24) Villanueva del Fresno	32 ± 6	0.10 ± 0.01	3 ± 1	0.30 ± 0.04	0.03 ± 0.00	0.42 ± 0.06	14 ± 1

Values of each determination represents mean ± SD (*n* = 3).

**Table 9:** Clusters center of gravity.

Variable	Cluster 1	Cluster 2	Cluster 3
Ag	2.433 ± 1.593	1.690 ± 1.248	1.690 ± 2.309
Al	396.503 ± 210.361	392.625 ± 226.842	227.812 ± 187.093
Ba	1.442 ± 0.962	0.996 ± 0.616	0.719 ± 0.616
Ca	583.148 ± 246.929	617.110 ± 151.838	413.341 ± 260.514
Cd	1.316 ± 1.120	0.854 ± 0.322	0.630 ± 0.526
Cr	1.213 ± 0.933	1.753 ± 1.122	0.656 ± 0.486
Cu	224.094 ± 168.207	238.065 ± 108.827	112.320 ± 90.198
Fe	63.880 ± 41.865	252.718 ± 74.423	36.283 ± 27.911
K	34192.173 ± 13478.898	23008.328 ± 23670.094	21750.972 ± 12089.189
Mg	864.049 ± 419.246	664.112 ± 268.732	533.816 ± 354.098
Mn	64.046 ± 29.957	76.347 ± 18.841	30.254 ± 19.582
Na	1240.238 ± 624.901	1286.973 ± 354.879	598.937 ± 391.228
P	422.333 ± 167.110	219.741 ± 66.174	182.971 ± 173.598
Pb	2.440 ± 1.468	3.389 ± 1.805	1.640 ± 1.256
Zn	72.936 ± 29.664	80.972 ± 33.836	45.118 ± 29.973

**Table 10:** The coincidence matrix for Decision Tree model presented in Figure 5.

Variables	Training set			Test set		
	Cluster 1	Cluster 2	Cluster 3	Cluster 1	Cluster 2	Cluster 3
Cluster 1	24	0	0	9	0	0
Cluster 2	0	8	0	0	4	0
Cluster 3	0	0	14	0	0	10

**Table 11:** The coincidence matrix for Decision Tree model presented in Figure 6.

Variables	Training Set			Test Set		
	Cluster 1	Cluster 2	Cluster 3	Cluster 1	Cluster 2	Cluster 3
Cluster 1	22	2	0	8	1	0
Cluster 2	1	7	0	1	3	0
Cluster 3	2	0	12	2	0	8

## References

[1] Agrahar-Murugkar D., Subbulakshmi G. (2005). Nutritional value of edible wild mushrooms collected from the Khasi hills of Meghalaya. *Food Chemistry*.

[2] De Pinho P. G., Ribeiro B., Gonçalves R. F. (2008). Correlation between the pattern volatiles and the overall aroma of wild edible mushrooms. *Journal of Agricultural and Food Chemistry*.

[3] Zawirska-Wojtasiak R., Siwulski M., Mildner-Szkudlarz S., Wa̧sowicz E. (2009). Studies on the aroma of different species and strains of Pleurotus measured by GC/MS, sensory analysis and electronic nose. *ACTA Scientiarum Polonorum Technologia Alimentaria*.

[4] Guillamón E., García-Lafuente A., Lozano M. (2010). Edible mushrooms: role in the prevention of cardiovascular diseases. *Fitoterapia*.

[5] Vetter J. (2005). Mineral composition of basidiomes of Amanita species. *Mycological Research*.

[6] Ouzouni P. K., Veltsistas P. G., Paleologos E. K., Riganakos K. A. (2007). Determination of metal content in wild edible mushroom species from regions of Greece. *Journal of Food Composition and Analysis*.

[7] Beluhan S., Ranogajec A. (2011). Chemical composition and non-volatile components of Croatian wild edible mushrooms. *Food Chemistry*.

[8] Reis F. S., Barros L., Martins A., Ferreira I. C. F. R. (2012). Chemical composition and nutritional value of the most widely appreciated cultivated mushrooms: an inter-species comparative study. *Food and Chemical Toxicology*.

[9] Wang X.-M., Zhang J., Li T., Wang Y.-Z., Liu H.-G. (2015). Content and bioaccumulation of nine mineral elements in ten mushroom species of the genus boletus. *Journal of Analytical Methods in Chemistry*.

[10] Falandysz J., Drewnowska M., Chudzińska M., Barałkiewicz D. (2017). Accumulation and distribution of metallic elements and metalloids in edible Amanita fulva mushrooms. *Ecotoxicology and Environmental Safety*.

[11] Chudzyński K., Falandysz J. (2008). Multivariate analysis of elements content of Larch Bolete (*Suillus grevillei*) mushroom. *Chemosphere*.

[12] Gençcelep H., Uzun Y., Tunçtürk Y., Demirel K. (2009). Determination of mineral contents of wild-grown edible mushrooms. *Food Chemistry*.

[13] Borovička J., Řanda Z., Jelínek E., Kotrba P., Dunn C. E. (2007). Hyperaccumulation of silver by Amanita strobiliformis and related species of the section Lepidella. *Mycological Research*.

[14] Borovička J., Řanda Z. (2007). Distribution of iron, cobalt, zinc and selenium in macrofungi. *Mycological Progress*.

[15] Kalač P. (2010). Trace element contents in European species of wild growing edible mushrooms: a review for the period 2000–2009. *Food Chemistry*.

[16] Falandysz J., Drewnowska M. (2015). Distribution of mercury in Amanita fulva (Schaeff.) Secr. mushrooms: Accumulation, loss in cooking and dietary intake. *Ecotoxicology and Environmental Safety*.

[17] Falandysz J. (2017). Mercury accumulation of three Lactarius mushroom species. *Food Chemistry*.

[18] Salvador C., Martins M. R., Vicente H., Neves J., Arteiro J. M., Caldeira A. T. (2013). Modelling molecular and inorganic data of Amanita ponderosa mushrooms using artificial neural networks. *Agroforestry Systems*.

[19] González V., Arenal F., Platas G., Esteve-Raventós F., Peláez F. (2002). Molecular typing of Spanish species of Amanita by restriction analysis of the ITS region of the DNA. *Mycological Research*.

[20] Moreno G., Platas G., Peláez F. (2008). Molecular phylogenetic analysis shows that Amanita ponderosa and A. curtipes are distinct species. *Mycological Progress*.

[21] Caldeira A. T., Salvador C., Pinto F., Arteiro J. M., Martins M. R. (2009). MSP-PCR and RAPD molecular biomarkers to characterize Amanita ponderosa mushrooms. *Annals of Microbiology*.

[22] Martins M. R., Salvador C., Vicente H., Neves J., Arteiro J. M., Caldeira A. T.

[23] Salvador C., Martins M. R., Arteiro J. M., Caldeira A. T. (2014). Molecular evaluation of some Amanita ponderosa and the fungal strains living in association with these mushrooms in the southwestern Iberian Peninsula. *Annals of Microbiology*.

[24] Caldeira A. T., Salvador C., Pinto F., Arteiro J. M., Martins M. R. Molecular biomarkers to characterize Amanita ponderosa mushrooms.

[25] Teresa Caldeira A., Arteiro J. M., Roseiro J. C., Neves J., Vicente H. (2011). An artificial intelligence approach to Bacillus amyloliquefaciens CCMI 1051 cultures: Application to the production of anti-fungal compounds. *Bioresource Technology*.

[26] Caldeira A. T., Roseiro J. C., Arteiro J. M., Neves J., Vicente H. (2011). Production of bioactive compounds against wood contaminant fungi: an artificial intelligence approach. *Minimizing the Environmental Impact of the Forest Products Industries*.

[27] Malençon G., Heim R. (1942). Notes critiques sur quelques hymΦnomycetes dEurope et dAfrique du Nord I. Les amanites blanches meridionales. *Bulletin trimestriel de la Société mycologique de France*.

[28] Moreno-Rojas R., Díaz-Valverde M. A., Arroyo B. M., González T. J., Capote C. J. B. (2004). Mineral content of gurumelo (Amanita ponderosa). *Food Chemistry*.

[29] Greenberg A. E., Eaton A. D. (1992). *Standard Methods for the Examination of Water and Wastewater*.

[30] Fernandes S. M. V., Rangel A. O. S. S., Lima J. L. F. C. (1997). Flow Injection Determination of Sodium, Potassium, Calcium, and Magnesium in Beer by Flame Emission and Atomic Absorption Spectrometry. *Journal of Agricultural and Food Chemistry*.

[31] Okumura F., Cavalheiro É. T. G., Nóbrega J. A. (2004). Simple flame photometric experiments to teach principles of atomic spectrometry in undergraduate analytical chemistry courses. *Química Nova*.

[32] AOAC, (Association of Official Analytical Chemist) Official methods of analysis, 15th ed, 2nd (suppl 991.25) 101–102, 1991

[33] MacQueen J. (1967). Some methods for classification and analysis of multivariate observations. *Proceedings of the 5th Berkeley Symposium on Mathematical Statistics and Probability*.

[34] Hall M., Frank E., Holmes G., Pfahringer B., Reutemann P., Witten I. H. (2009). The WEKA data mining software: an update. *ACM SIGKDD Explorations Newsletter*.

[35] Witten I. H., Frank E., Hall M. A. (2011). *Data Mining - Practical Machine Learning Tools and Techniques*.

[36] Kalač P., Svoboda L. (2000). A review of trace element concentrations in edible mushrooms. *Food Chemistry*.

[37] Rudawska M., Leski T. (2005). Macro- and microelement contents in fruiting bodies of wild mushrooms from the Notecka forest in west-central Poland. *Food Chemistry*.

[38] Sesli E., Tuzen M., Soylak M. (2008). Evaluation of trace metal contents of some wild edible mushrooms from Black sea region, Turkey. *Journal of Hazardous Materials*.

[39] European Commission, European Commission Opinion of the Scientific Committee on Food on the Tolerable Upper Intake Level of Copper. Health and Consumer Protection Directorate-General, Brussels, Belgium, 2003

[40] FAO/WHO, Human vitamin and mineral requirements. World Health Organization, Food and Agriculture Organization of United Nations, Rome, Italy, 2002

[41] Sesli E., Tüzen M. (1999). Levels of trace elements in the fruiting bodies of macrofungi growing in the East Black Sea region of Turkey. *Food Chemistry*.

[42] Afzali D., Mostafavi A., Taher M. A., Moradian A. (2007). Flame atomic absorption spectrometry determination of trace amounts of copper after separation and preconcentration onto TDMBAC-treated analcime pyrocatechol-immobilized. *Talanta*.

[43] Soriano S., Netto A. D. P., Cassella R. J. (2007). Determination of Cu, Fe, Mn and Zn by flame atomic absorption spectrometry in multivitamin/multimineral dosage forms or tablets after an acidic extraction. *Journal of Pharmaceutical and Biomedical Analysis*.

[44] Abbaspour N., Hurrell R., Kelishadi R. (2014). Review on iron and its importance for human health. *Journal of Research in Medical Sciences*.

[45] Mendil D., Uluözlü Ö. D., Hasdemir E., Çağlar A. (2004). Determination of trace elements on some wild edible mushroom samples from Kastamonu, Turkey. *Food Chemistry*.

[46] Gerber G. B., Léonard A., Hantson P. (2002). Carcinogenicity, mutagenicity and teratogenicity of manganese compounds. *Critical Review in Oncology/Hematology*.

[47] Tuzen M., Sesli E., Soylak M. (2007). Trace element levels of mushroom species from East Black Sea region of Turkey. *Food Control*.

[48] Ma J. F., Ryan P. R., Delhaize E. (2001). Aluminium tolerance in plants and the complexing role of organic acids. *Trends in Plant Science*.

[49] Cocchi L., Vescovi L., Petrini L. E., Petrini O. (2006). Heavy metals in edible mushrooms in Italy. *Food Chemistry*.

[50] Souza J., Japkowicz N. Evaluating data mining models: a pattern language.

[51] Provost F., Kohavi R. (1998). Guest editors' introduction: On applied research in machine learning. *Machine Learning*.

